# Factor XIII polymorphism and risk of aneurysmal subarachnoid haemorrhage in a south Indian population

**DOI:** 10.1186/s12881-018-0674-x

**Published:** 2018-09-05

**Authors:** Arati Suvatha, M. K. Sibin, Dhananjaya I. Bhat, K. V. L. Narasingarao, Vikas Vazhayil, G. K. Chetan

**Affiliations:** 1 Department of Human Genetics, National Institute of Mental Health and Neuro Sciences, Bangalore, Karnataka 560029 India; 2 Department of Biochemistry, Armed Forces Medical College, Pune, 411040 India; 3 Department of Neurosurgery, National Institute of Mental Health and Neuro Sciences, Bangalore, 560029 India

**Keywords:** Aneurysmal subarachnoid haemorrhage, Factor XIII, Polymorphism, Basilar top aneurysm

## Abstract

**Background:**

The rupture of a brain aneurysm causes bleeding in the subarachnoid space and is known as aneurysmal subarachnoid haemorrhage (aSAH). In our study, we evaluated the association of *factor XIII* polymorphism and the risk of Aneurysmal subarachnoid haemorrhage (aSAH) in South Indian population.

**Methods:**

The study was performed in 200 subjects with aSAH and 205 healthy control subjects. Genotyping of rs5985(c.103G > T (p.Val35Leu)) and rs5982(c.1694C > T (p.Pro564Leu)) polymorphism was performed by Taqman® allelic discrimination assay.

**Results:**

In our study, Val/Leu genotype frequency was higher in control subjects (18%) compared to aSAH patients (9%).The Val/Leu genotype was associated with lower risk of aSAH (OR = 0.48, 95%CI = 0.26–0.88, *p* = 0.02). When compared with Val allele, Leu allele was significantly associated with lower risk of aSAH (OR = 0.55, 95%CI = 0.32–0.95, *p* = 0.03). In subtyping, we found a significant association of Leu/Leu genotype with the Basilar top aneurysm (OR = 3.59, 95%CI = 1.11–11.64, *p* = 0.03). In c.1694C > T (p.Pro565Leu) variant, Pro/Pro Vs Pro/Leu genotype (OR = 2.06, 95%CI = 1.10–3.85, *p* = 0.02) was significantly associated with higher risk of aSAH. The 564Leu allelic frequency in aSAH patients (36%) was higher when compared with that in healthy controls (30%) in our study. When allele frequency (Pro Vs Leu) was compared, 564Leu allele was found to be significantly associated with higher aSAH risk (OR = 1.36, 95%CI = 1.01–1.83, *p* = 0.04). (OR = 1.36, 95%CI = 1.01–1.83, *p* = 0.04). Regarding rs5985 and rs5982, significant association was found in the log-additive model (OR = 0.57, 95%CI = 0.33–0.97, *p* = 0.034; OR = 1.32, 95%CI = 1.00–1.72, *p* = 0.043).

**Conclusion:**

These results suggest that 34Leu allele was a protective factor for lower risk of aSAH whereas 564Leu allele was associated with higher risk of aSAH in South Indian population.

**Electronic supplementary material:**

The online version of this article (10.1186/s12881-018-0674-x) contains supplementary material, which is available to authorized users.

## Background

Subarachnoid hemorrhage (SAH) caused by rupture of a cerebral aneurysm is the reason for approximately 85% of cases with spontaneous SAH [1]. It accounts for 5% of all stroke cases and is associated with high rate of mortality and morbidity [2]. Rebleeding and delayed cerebral ischemia are the two major complications that are associated with poor prognosis and high mortality rate in SAH [3]. The first-degree relatives of patients with SAH have a three-fold increased risk for the rupture of an aneurysm when compared with general population [4]. But the role of genetic factors which contribute to the risk of SAH is poorly defined. Most candidate gene studies have considered proteins associated with connective tissue organization [5–7]. The reason for SAH occurrence was not only due to weakened vessel wall structure but also due to rupture of vessel wall [8]. A few studies have investigated the role of fibrinolytic system and coagulation factors association with the risk of aSAH [9–11].

Coagulation factor XIII belongs to transglutaminase family which circulates as a heterotetramer, composed of two A subunits and two B subunits [12]. During coagulation, thrombin activates the catalytic factor XIII A subunit and crosslinks the fibrin molecules to increase the clot stability [13]. During fibrinolysis, factor XIII A activates anti plasmin which inhibits the plasmin from degrading the crosslinked fibrin structure [14]. Thus, factor XIII A subunit plays a significant role both in coagulation and fibrinolysis. Also, it plays a key role in extracellular remodelling, angiogenesis, atherosclerosis, wound healing and tissue repair [15].

In humans, the Coagulation factor XIII A chain (*F13A*) gene is located on chromosome 6p 24–25 [16]. The factor XIII A is 83 kDa protein, which consists of 732 amino acids [17]. *F13A* gene consists of 15 exons and 14 introns [18]. The nine polymorphisms in *F13A* genes are c.103G > T(p.Val35Leu), c.614A > T(p.Tyr204Phe), c.996A > C (p.Pro332Pro), c.1652C > T(p.Thr550Ile), c.1694C > T (p.Pro564Leu), c.1704A > G (p.Glu567Glu), c.1696 T > A (p.Leu588Gl), c.1951G > A (p.Val650Ile) and c.1954G > C (p.Glu652Gln) [19]. Among them, the common *F13A* polymorphisms are c.103G > T (p.Val35Leu)and c.1694C > T (p.Pro564Leu).

In the Asian and Caucasian population, the allele frequency of 34Leu allele is 0.13 and 0.25 [20]. In Han Chinese population, the c.103G > T (p.Val35Leu) polymorphism was associated with the risk of ischemic cardiovascular and cerebrovascular diseases [21].In Caucasian population, c.103G > T (p.Val35Leu) polymorphism was associated with the risk of intracerebral hemorrhage and brain infarction [22, 23].In the Asian and Caucasian population, the allele frequency of 564Leu allele is 0.29 and 0.21 [17]. The c.1694C > T(p.Pro564Leu) polymorphism was associated with decreased factor XIII plasma levels with increased factor XIII activity [24]. When stratified by gender c.1694C > T (p.Pro564Leu) polymorphism was associated with risk of haemorrhagic stroke in women aged < 45 years in Caucasian population [10]. The aim of the present study is to investigate the association of c.103G > T (p.Val35Leu) and c.1694C > T (p.Pro564Leu) polymorphisms with the risk of aSAH in a South Indian population.

## Methods

### Study population

A total of 200 patients with aneurysmal subarachnoid haemorrhage and 205, age and sex- matched healthy controls were selected randomly from general population during the period of 2015–2017. The healthy controls were unrelated to patients but were of the same ethnicity. Also, patients were unrelated to each other. The patients were recruited from the Department of Neurosurgery, NIMHNAS, Bangalore, India and their demographic and clinical details were collected from the medical records department of the hospital. The neurological grade was classified based on World Federation of neurological surgeons (WFNS) scale and all grades were included in this study. The inclusion criteria for selecting patients with aSAH was the presence of symptoms suggestive of aSAH combined with the finding of subarachnoid blood on CT and a proven aneurysm on conventional angiography. Exclusion criteria for selecting patients were 1.the presence of neuropsychiatric conditions like dementia, Parkinson’s disease, epilepsy, psychoses 2. SAH resulted from a mycotic aneurysm, arterio-venous malformation, or head trauma. The inclusion criteria for healthy controls were 1. the absence of clinical symptoms of aSAH 2. similar demographic characteristics of patients such as adult group over 18 years old, gender, ethnicity and dietary habits, 3. no medical history of haemorrhage and no family history of aSAH in first degree relatives. The study protocol was approved by the Institute of ethics committee for human studies, NIMHANS, Bangalore. Written informed consent was obtained from all the participants included in the study.

### DNA extraction and genotyping

Five milliliter blood sample was collected from all the participants and genomic DNA was isolated from blood using commercially available Machery-Nagel (MN) kit according to manufacturer’s protocol. DNA with a purity of 1.75–1.85 was used for genotyping analysis. Purity and quantity of DNA was analysed by Nanodrop ND2000c spectrophotometer. Genotyping of c.103G > T (p.Val35Leu) (rs5985) and c.1694C > T (p.Pro564Leu) (rs5982) was performed using Taqman® allelic discrimination assay (Applied Biosystems, Foster City, CA) with a commercially available primer probe set (assay ID C_1639938_20, C_8786720_10). Experiments were performed in duplicates in Applied Biosystem7500 Fast real-time machine.

### Statistical analysis

R.3.0.11 statistical software was used to statistically analyse the data. The continuous variables were expressed as mean ± SD and categorical variables were expressed as absolute values and percentages. The difference in genotype and allele frequencies between groups were analysed by χ2 test. Association between *F13A* genotypes or alleles and aSAH risk were expressed as odds ratio (OR) with 95% confidence intervals (CI), adjusted for the confounding effects of smoking, hypertension, drinking and diabetes mellitus using the logistic regression model. *p*-value < 0.05 was considered significant. The Hardy-Weinberg equilibrium calculation and additive effect of SNPs was calculated using the online tool SNPStats, https://www.snpstats.net/start.html [25]. Prediction of functional effect of two SNPs mapped in genetic variants of *F13A* gene was done using SIFT (http://sift.jcvi.org/) and PolyPhen-2 (http://genetics.bwh.harvard.edu/pph/data/index.html) [26].The linkage disequilibrium (LD) and haplotype frequency were estimated using Haploview software (version 4.2). The meta-analysis study was performed for fixed and random effect model using Review manager5.2. The test for heterogeneity was estimated by I^2^ statistics. *p-*value < 0.10 was considered as significant for heterogeneity among the studies. Fixed effect model was used to find out the OR with 95%CI when there was no heterogeneity; otherwise, random effect model was applied [27]. Val/Val and Pro/Pro genotypes were the wild- type homozygote genotype for *F13A* gene, while Leu/Leu genotype was the rare homozygous genotype. The dominant and recessive models for this study were Val/Val Vs Val/Leu + Leu/Leu, Pro/Pro Vs Pro/Leu + Leu/Leu, Val/Val + Val/Leu Vs Leu/Leu and Pro/Pro+ Pro/Leu Vs Leu/Leu.

## Results

### Characteristics of study population

Demographic characteristics of aSAH patients and controls were already published previously (DOI: 10.1186/s11658-017-0059-8). There were no significant differences in gender and mean age between aSAH patients and healthy controls.

### *Factor XIII* polymorphism and risk of aSAH

The distribution of *factor XIII* genotype and allele frequencies is shown in Table 1.The distribution of genotype frequencies of controls are in Hardy–Weinberg equilibrium (rs5985; *p* = 0.99, rs5982; *p* = 0.79). In our study, for c.103G > T(p.Val35Leu) and c.1694C > T (p.Pro564Leu) variants there was no significant difference in genotypes (χ^2^ = 5.81; df = 2; *p* = 0.05); (χ^2^ = 5.41; df = 2; *p* = 0.06) between cases and controls. However, in allele frequencies (χ^2^ = 4.12; df = 1; *p* = 0.04); (χ^2^ = 3.89; df = 1; *p* = 0.04) there was a significant difference for c.103G > T (p.Val35Leu) and c.1694C > T (p.Pro564Leu) variants between cases and controls.

**Table 1:** Genotypes and allele frequency of *F13A* polymorphisms in aSAH Cases and Controls

	Alleles		Genotypes		
	*n*(%)	*n*(%)	*n*(%)	*n*(%)	*n*(%)
c.103G > T (p.Val35Leu)	Val	Leu	Val/Val	Val/Leu	Leu/Leu
Control subjects (205)	371(93)	39(10)	168(84)	35(17.5)	2(1)
aSAH patients (200)	378(95)	22(6)	180(90)	18(9)	2(1)
c.1694C > T (p.Pro564Leu)	Pro	Leu	Pro/Pro	Pro/Leu	Leu/Leu
Control subjects (205)	292(73)	118 (30)	106(53)	80(40)	19(9.5)
aSAH patients (200)	258(65)	142(36)	92(46)	74(37)	34(17)

The result of logistic regression analyses is shown in Table 2 and Additional file 1: Table S1. In c.103G > T (p.Val35Leu) variant, the Val/Leu genotype frequency was higher in control subjects (18%) when compared with that in aSAH patients (9%).The presence of one copy of 34Leu allele was associated with lower risk of aSAH (Val/Val Vs Val/Leu; OR = 0.45, 95%CI = 0.24–0.84; *p* = 0.013). In the dominant model of inheritance, there was a significant association between c.103G > T (p.Val35Leu) polymorphism and risk of aSAH (Val/Val Vs Val/Leu + Leu/Leu; OR = 0.48, 95%CI = 0.26–0.84; *p* = 0.013). However, the presence of two copies of 34Leu allele was not significantly associated with aSAH risk (Val/Val Vs Leu/Leu; OR = 1.19, 95%CI = 0.16–8.65; *p* = 0.858).Likewise, the recessive model of c.103G > T (p.Val35Leu) polymorphism did not have any statistical significance. A significant association was found in the log-additive model for rs5985 (c.103G > T (p.Val35Leu)) with an OR of 0.57 (95% CI = 0.33–0.97; *p* = 0.034). In our study, the 34Leu allelic frequency in healthy controls subjects (10%) was higher than that in aSAH patients (6%). When allele frequency (Val Vs Leu) was compared, 34Leu allele was significantly associated with lower aSAH risk (OR = 0.55, 95%CI = 0.32–0.95; *p* = 0.030).

**Table 2:** Logistic Regression Analysis of association between *F13A* SNPs and aSAH risk

Genotype & Allele	Adjusted OR^a^ (95%CI)	*p-*value
c.103G > T (p.Val35Leu) Model		
Dominant	0.48(0.26–0.89)	**0.020**
Recessive	1.30(0.18–9.41)	0.791
Log-Additive model	0.57 (0.33–0.97)	**0.034**
c.1694C > T (p.Pro564Leu) Model		
Dominant	1.25(0.83–1.86)	0.275
Recessive	1.94(1.05–3.58)	**0.034**
Log-Additive model	1.32 (1.00–1.72)	**0.043**

*OR* Odds Ratio

^a^Adjusted for smoking, alcohol consumption, hypertension and diabetes

*p*-values < 0.05 are given in bold

In c.1694C > T (p.Pro564Leu) variant, the Leu/Leu genotype was higher in aSAH patients (17%) when compared with that in healthy controls (9.5%). The presence of two copies of the 564Leu allele was significantly associated with higher risk of aSAH (Pro/Pro Vs Leu/Leu; OR = 2.00, 95%CI = 1.15–3.76; *p* = 0.034).Also, in the recessive model of inheritance, there was a significant association between c.1694C > T (p.Pro564Leu) polymorphism and risk of aSAH (Pro/Pro+ Pro/Leu Vs Leu/Leu; OR = 1.94, 95%CI = 1.05–3.58; *p* = 0.034). Similarly, a significant association was found in the log-additive model for rs5982 (c.1694C > T (p.Pro564Leu)) with an OR of 1.32 (95% CI = 1.00–1.72; *p* = 0.043). Our studies showed that 564Leu allelic frequency in aSAH patients (36%) was higher than that in healthy controls (30%). When allele frequency (Pro Vs Leu) was compared, 564Leu allele was significantly associated with higher aSAH risk (OR = 1.36, 95%CI = 1.01–1.83; *p* = 0.040). However, there was no significant association in heterozygous genotype and dominant model of inheritance with the risk of aSAH.

When aneurysm was classified according to the location, size and WFNS grade, only the Leu/Leu genotype in c.103G > T (p.Val35Leu) variant was statistically significant with basilar top aneurysm (OR = 3.59, 95%CI = 1.11–11.64; *p* = 0.030). Classification of aneurysm according to c.103G > T (p.Val35Leu) and c.1694C > T (p.Pro564Leu) variants is shown in Table 3. Multiple comparisons were performed between male versus female, hypertensive versus non-hypertensive, diabetic versus non- diabetic patients with different c.103G > T (p.Val35Leu) and c.1694C > T (p.Pro564Leu) genotypic model and allele frequencies. None of the comparison showed statistical significance with c.103G > T (p.Val35Leu) and c.1694C > T (p.Pro564Leu) variants.

**Table 3:** c.103G > T (p.Val35Leu) and c.1694C > T (p.Pro564Leu) Variants in aSAH subtypes

Variable	Case	Val/Val (*p*)	Val/Leu (*p*)	Leu/Leu (*p*)	Val	Leu	Val Vs Leu *(p)*	Pro/Pro (*p*)	Pro/Leu (*p*)	Leu/Leu (*p*)	Pro	Leu	Pro Vs Leu *(p)*
Total	200	180	18	2	378	22		92	74	34	258	142	
Site of Aneurysm													
ACOM	86	75(0.86)	11(0.38)	0(0.62)	161	11	0.67	42 (0.79)	26 (0.44)	18(0.51)	110	62	0.90
PCOM	12	11(0.96)	1(0.94)	0(0.45)	23	1	0.78	4 (0.58)	7(0.35)	1(0.50)	15	9	0.84
ICA	36	32(0.96)	3(0.90)	1(0.40)	67	5	0.62	16(0.91)	16(0.57)	4(0.44)	48	24	0.72
MCA	37	36(0.76)	1(0.24)	0(0.96)	73	1	0.16	14 (0.56)	17(0.50)	6(0.92)	45	29	0.54
Multiple	22	20(0.97)	2(0.98)	0(0.71)	42	2	0.79	13 (0.50)	6(0.52)	3(0.73)	32	12	0.27
Basilar top	7	6(0.93)	0(0.82)	1(**0.03)**	12	2	0.18	3 (0.91)	2(0.75)	2(0.52)	8	6	0.57
Size of aneurysm													
Small(< 15 mm)	159	141(0.92)	17(0.62)	1(0.70)	299	19	0.78	76(0.83)	55(0.74)	28(0.89)	207	111	0.86
Large(15-25 mm)	37	36(0.76)	0	1(0.42)	72	2	0.32	15(0.70)	17(0.50)	5(0.65)	47	27	0.87
Giant(> 25 mm)	4	3(0.81)	1(0.37)	0	7	1	0.41	1(0.58)	2 (0.73)	1(0.73)	4	4	0.40
WFNS Grade													
Grade I	91	82(0.99)	8(0.95)	1(0.93)	172	10	0.99	38(0.67)	31(0.73)	22(0.24)	107	75	0.18
Grade II	42	36(0.84)	5(0.59)	1(0.48)	77	7	0.32	21(0.77)	17(0.77)	4(0.29)	59	25	0.31
Grade III	51	47(0.91)	4(0.81)	0	98	4	0.52	26(0.71)	20(0.40)	5(0.27)	72	30	
Grade IV	16	15(0.91)	1(0.73)	0	31	1	0.57	7(0.91)	6(0.97)	3(0.88)	20	12	0.82
Male	77	73(0.78)	4(0.33)	0	150	4	0.15	39(0.68)	26(0.72)	12(0.81)	104	50	0.50
Female	123	107(0.14)	14(0.53)	2(0.62)	228	18	0.35	53(0.75)	48(0.80)	22(0.86)	154	92	0.62
Hypertension (+)	37	32(0.33)	5(0.44)	0	69	5	0.66	14(0.56)	18(0.38)	5(0.65)	46	28	0.70
Diabetes mellitus (+)	71	65(0.93)	5(0.63)	1(0.78)	136	7	0.78	34(0.86)	25(0.85)	12(0.98)	93	49	0.83
Alcohol (+)	57	53(0.88)	4(0.66)	0	110	4	0.39	29(0.69)	18(0.60)	10(0.93)	76	38	0.66
Smoking (+)	58	54(0.87)	4(0.64)	0	112	4	0.37	31(0.55)	19(0.68)	8(0.61)	81	35	0.28

*p*-value < 0.05 are given in bold

Prediction of the functional effect of studied SNPs was done with two annotation programs, namely SIFT (Sorting Intolerant from Tolerant) and PolyPhen-2 (Polymorphism Phenotyping). Using SIFT algorithm, the normalized probability score for rs5985 and rs5982 was > 0.05 (1 and 0.14) and predicted to be tolerated. Using PolyPhen-2 algorithm, the normalized probability score for rs5985 and rs5982 was < 0.2 (0 and 0.003) and predicted as benign. According to the sequence and structural homology-based approach, the studied nsSNPs has tolerated/benign functional prediction score (Additional file 2: Table S2).

### Linkage disequilibrium (LD) and haplotype analysis of *factor XIII* and aSAH

**Fig. 1:**
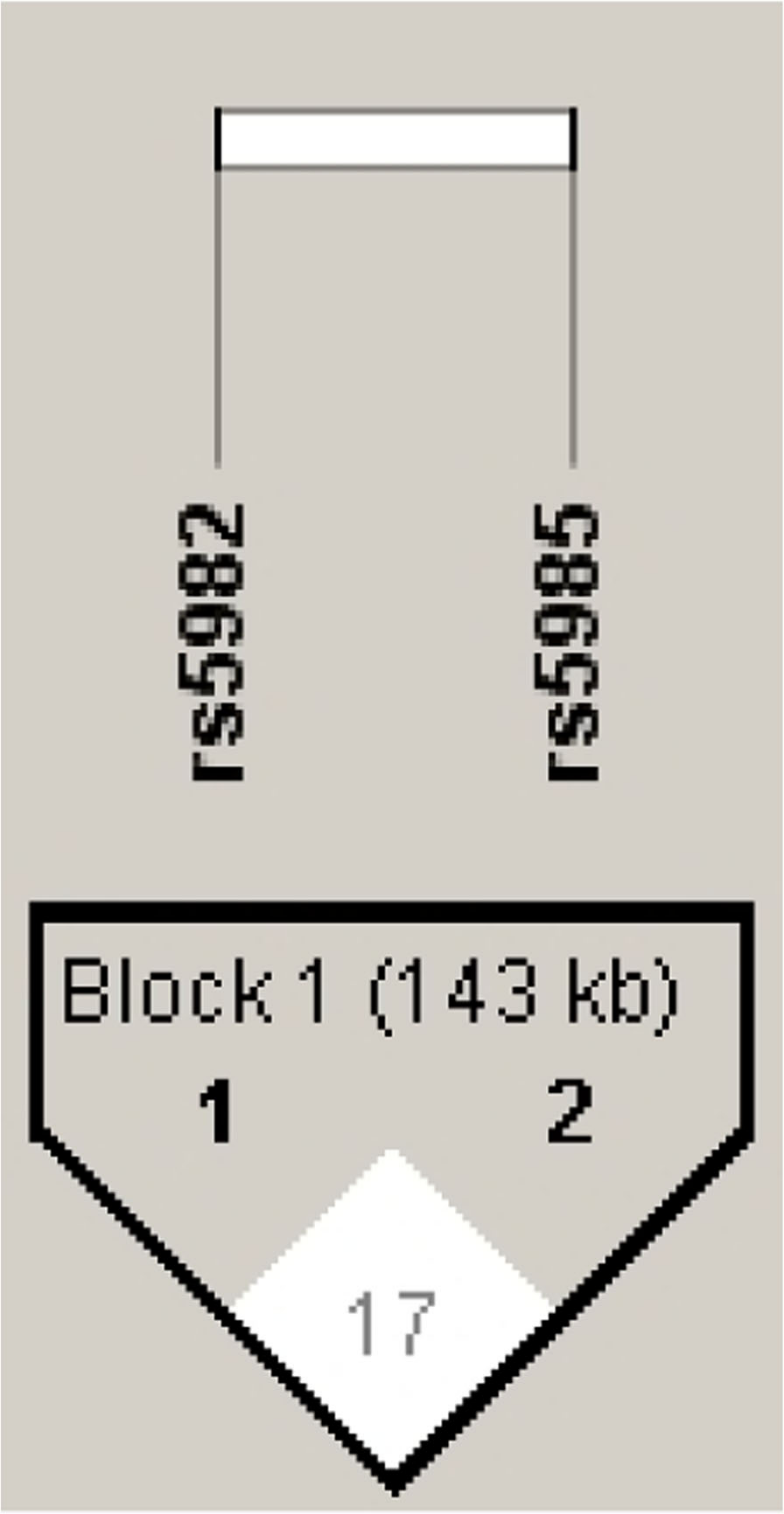
LD pattern of *factor XIII* polymorphism

Haploview software was used to estimate the LD between the two-studied polymorphism. There was no significant LD (D’ = 0.17) observed among the polymorphism (Fig. 1), which suggest the strongest evidence of recombination. The haplotype frequency estimation among patients and controls is shown in Table 4. The frequency of Leu:Val haplotype (c.1694C > T (p.Pro564Leu): c.103G > T (p.Val35Leu)) was significantly higher in controls than in aSAH patients (*p* = 0.01). Whereas the frequency of Pro:Leu (c.1694C > T (p.Pro564Leu): c.103G > T (p.Val35Leu)) haplotype was significantly higher in aSAH patients than in controls (*p* = 0.03).Pro:Val (c.1694C > T (p.Pro564Leu): c.103G > T (p.Val35Leu)) was the most frequent haplotype and was observed in more than 60% in both aSAH patients and controls.

**Table 4:** Haplotype frequency distribution among patients and controls

Haplotypes	Frequencies in controls (%)	Frequencies in patients (%)	χ2	*p*
Pro:Val	60.6	64.1	1.08	0.29
Leu:Val	33.9	26.1	5.86	0.01
Pro:Leu	3.9	7.3	4.47	0.03
Leu:Leu	1.6	2.4	0.71	0.40

Order of SNPs in *F13A* haplotypes: c.1694C > T (p.Pro564Leu), c.103G > T (p.Val35Leu); χ2: Chi-square test; *p*: probability value

### Meta-analysis of *factor XIII* polymorphism with risk of aSAH

**Fig. 2:**
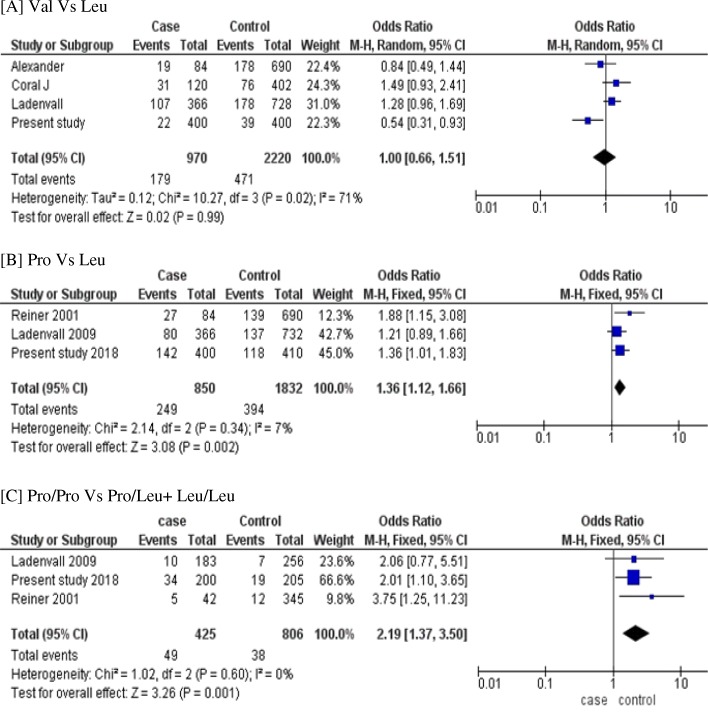
Metanalysis of *factor XIII* gene variants. **a** c.103G > T (p.Val35Leu) (Val Vs Leu) (**b**) c.1694C > T (p.Pro564Leu) (Pro Vs Leu) (**c**) c.1694C > T (p.Pro564Leu) (Pro/Pro Vs Pro/Leu + Leu/Leu)

We performed the meta-analysis with previously reported studies along with our present study to verify the association between *F13A* gene polymorphism and risk of aSAH. The meta -analysis of c.103G > T (p.Val35Leu) variant could not predict any significant association with aSAH risk in fixed effect and random effect models. There was significant heterogeneity in Val Vs Leu (*p* = 0.02) and in dominant model (*p* = 0.03). However, in c.1694C > T (p.Pro564Leu) variant there was significant association in Pro Vs Leu allele, Pro/Pro Vs Leu/Leu genotype and in dominant model of inheritance (Pro Vs Leu, OR = 1.36, 95%CI =1.12–1.66; *p* = 0.002; Pro/Pro Vs Leu/Leu, OR = 2.49, 95%CI =1.53–4.06; *p* = 0.0002; Pro/Pro Vs Pro/Leu + Leu/Leu, OR = 2.19, 95%CI = 1.37–3.50; *p* = 0.001) (Fig. 2).

## Discussion

Spontaneous subarachnoid hemorrhage (non-traumatic) remains as one of the considerable neurosurgical problems that affect 25,000 to 28,000 people yearly [28]. In cerebrovascular disorders, the role of multifactorial and multigene have been studied progressively. The difference in phenotype in persons carrying same genetic mutation suggests the role of multiple factors in the pathogenesis of the disease [29]. This study was carried out to analyse whether *F13A* polymorphism was associated with the risk of aSAH.

Extracellular matrix remodelling dysfunction, atherosclerosis and fibrinolytic dysfunction were considered as important pathogenic mechanisms in the formation and rupture of a cerebral aneurysm [30–32]. Coagulation factor XIII A chain plays a significant role in extracellular matrix (ECM) remodelling and tissue repair [33]. Crosslinking of collagen and fibronectin to each other by F13A during extracellular matrix formation and wound healing was an important physiological event in stabilizing the ECM [34]. F13A in the cellular form plays a significant role in triggering atherosclerosis [18]. F13A helps in angiotensin I receptor dimerization which activates the monocyte adhesion to endothelium cells and this was considered as one of the pathogenic mechanism in the progression of atherosclerosis [33]. In the fibrinolytic system, the primary mechanism to prolong fibrinolysis is crosslinking of α_2_ -anti plasmin and fibrin by F13A [35]. It has been shown that properties of F13A were affected by its gene variants [19] and it was suggested that *F13A* variants play a key role in the pathogenesis of a cerebral aneurysm by affecting the vessel wall stability, triggering atherosclerosis and decreasing clot stability [20].

*F13A* polymorphism was associated with the severity of outcome in atherothrombotic ischemic stroke [36], primary intracerebral hemorrhage [37] brain infarction [38] and deep vein thrombosis [39]. Many case-control studies reported the association of *F13A* polymorphism and risk of aSAH. Ladenvall et al. reported that 34Leu and 564Leu carriers had an increased risk of aSAH in the Swedish population [9], but there was no association between c.103G > T (p.Val35Leu) variant and nonfatal haemorrhagic stroke in young white women in U.S population [10]. Another study done by Rugriok et al. reported that c.103G > T (p.Val35Leu) and c.1694C > T (p.Pro564Leu) polymorphisms did not have any association with the risk for aSAH in Caucasian population [11]. In Spanish population, the prevalence of 34 Leu allele was higher in aSAH than in primary intracerebral hemorrhage group [40]. The meta-analysis of four studies including the present study suggested that there was no significant association with c.103G > T (p.Val35Leu) polymorphism and risk of aSAH, whereas the c.1694C > T (p.Pro564Leu) polymorphism showed significant association with risk of aSAH.

The c.103G > T (p.Val35Leu) polymorphism present at exon 2 of *F13A* gene increases the activation rate of coagulation and affects the fibrin structure [41]. The fibrin clot which is crosslinked by 34Leu variants has thinner fibres, smaller pore and altered permeation characteristics when compared with fibrin clot crosslinked by Val34 variant [19]. Also, the clot formation time was shorter for 34Leu variant samples [42]. The c.1694C > T (p.Pro564Leu) polymorphism present at exon 12 affects the specific activity of the enzyme. Also, c.1694C > T (p.Pro564Leu) variant causes lower plasma F13A levels and increases F13A activity [43]. In the present study, 34Leu allele was associated with lower risk and 564Leu allele was associated with the higher risk for aSAH.

The protective effect of the *F13A* c.103G > T (p.Val35Leu) polymorphism is not well understood and needs to be elucidated. The protective effect c.103G > T (p.Val35Leu) polymorphism was reported in few studies on myocardial infraction and venous thrombosis [44–47]. .An increased F13A activity was reported in 34Leu carriers, higher activity in Leu homozygotes and intermediate activity in Leu heterozygote [43]. This was because of proximity of polymorphism to the thrombin activation site. Kohler et al. reported that the higher F13A activation results in ineffective cross linking [48]. Van Wersch et al. reported that in pregnant women, F13A levels were higher in smokers than in non -smokers [49]. In our study number of smokers in patients were higher than that in controls. Elbaz et al. reported that the ORs associated with smoking were lower in 34Leu carriers than in noncarriers. This suggests that the protective effect of polymorphism was more significant than effect of smoking in 34Leu carriers [22]. The investigation of F13A activity in healthy controls while taking the effect of smoking and c.103G > T (p.Val35Leu) polymorphism in to account will be helpful for better understanding.

Basilar top aneurysm is the most common aneurysm seen in the posterior fossa circulation. It was characterised with higher bleeding tendency and worst clinical outcome after rupture [50]. In this study, 42.8% of patients with basilar top aneurysm had WFNS grade 1 and 71.4% of patients had WFNS grade 2 and 3. Therefore, most of the patients with basilar top aneurysm had the worst clinical outcome in this study.34Leu variant affects clot stability and thereby associated with the bleeding tendency [51]. Basilar top aneurysm was characterised by bleeding tendency and this explains the reason for the association between Leu/Leu genotype and basilar top aneurysm in this study.

The SIFT algorithm predicts the ‘damaging’ and ‘non-damaging’ (tolerated) SNPs based on the sequence homology and physical properties of sequence submitted [52]. The PolyPhen-2 algorithm predicts the nsSNPs in three distinct categories: ‘probably damaging’, ‘possibly damaging’ and ‘benign’ SNPs based on the structural homology-based approach using functional point of view [53]. The SNPs predicted as damaging /deleterious in both sequence and structural homology-based approach are considered as ‘high-confidence’ nsSNP, since they have higher impact on the function of protein [52, 54]. The rs5985 and rs5982 SNPs do not have any direct structural-functional effect on factor XIII A protein according to SIFT and PolyPhen-2 annotation programs. But the studied SNPs might have effect on factor XIII A protein through other indirect pathway.

There are previous reports of linkage disequilibrium (LD) between the variants of *F13A* gene [9, 10]. LD is the non-random association of alleles in two or more loci [55]. LD block (haplotype) is clinically important for the identification of disease causing genes and the origin of mutations [56]. Haplotypes occurs when SNPs are situated near to each other in the chromosome and are inherited in blocks [57]. In both the haplotypes, we found a significant association with the risk of aSAH. Haplotypes are more powerful than individual polymorphism for detecting susceptibility alleles associated with diseases [56, 57].

## Conclusion

Our study established that 34Leu carriers are associated with a lower risk and 564Leu carriers are association with a higher risk of aSAH in South Indian population. To the best of our knowledge, this is the first case-control study that has reported the association of *F13A* polymorphism with the risk of aSAH in South Indian population. Larger studies are required from other ethnic populations to determine the association of *factor XIII* polymorphism with the risk of aSAH, especially in the subtypes.

## Additional files

Additional file 1: **Table S1.** Odds ratios for risk of aneurysmal subarachnoid hemorrhage according to genotype and allele. (DOCX 15 kb)

Additional file 2: **Table S2.** Prediction of functional effect of studied SNPs. (DOCX 14 kb)

## Abbreviations

ACOM: Anterior communicating artery
aSAH: aneurysmal subarachnoid haemorrhage
CI: Confidence interval
F13A: Factor XIII A subunit
ICA: Internal carotid artery
MCA: Middle cerebral artery
OR: Odds ratio
PCOM: Posterior communicating artery
WFNS: World Federation of Neurological Surgeons
