# Supplementary material for: Factor XIII polymorphism and risk of aneurysmal subarachnoid haemorrhage in a south Indian population
Source: BMC Med Genet. 2018 Sep 5;19:159. doi: 10.1186/s12881-018-0674-x (PMC6126001; doi:10.1186/s12881-018-0674-x)
Supplement: Supplementary file 1 — Table S1. Odds ratios for risk of aneurysmal subarachnoid hemorrhage according to genotype and allele. (DOCX 15 kb) [file 12881_2018_674_MOESM1_ESM.docx]

| **Genotype & Allele** | **Adjusted OR† (95%CI)** | ***p-*value** |
| --- | --- | --- |
| **c.103G>T (p.Val35Leu)** |  |  |
| Val/Val Vs Leu/Leu | 1.19(0.16-8.65) | 0.858 |
| Val/Val Vs Val/Leu | 0.45(0.24-0.84) | **0.013** |
| Val Vs Leu | 0.55(0.32-0.95) | **0.030** |
| **c.1694C>T (p.Pro564Leu)** |  |  |
| Pro/Pro Vs Leu/Leu | 2.00(1.05-3.79) | **0.034** |
| Pro/Pro Vs Pro/Leu | 1.06(0.69-1.64) | 0.761 |
| Pro Vs Leu | 1.36(1.01-1.83) | **0.040** |

**Table S1.** Odds ratios for risk of aneurysmal subarachnoid hemorrhage according to genotype and allele

OR: Odds Ratio

†Adjusted for smoking, alcohol consumption, hypertension and diabetes

*p*-values <0.05 are given in bold.
