# Supplementary material for: Factor XIII polymorphism and risk of aneurysmal subarachnoid haemorrhage in a south Indian population
Source: BMC Med Genet. 2018 Sep 5;19:159. doi: 10.1186/s12881-018-0674-x (PMC6126001; doi:10.1186/s12881-018-0674-x)
Supplement: Supplementary file 2 — Table S2. Prediction of functional effect of studied SNPs. (DOCX 14 kb) [file 12881_2018_674_MOESM2_ESM.docx]

| SNP rsID | SNP type | SIFT Prediction | | PolyPhen-2. Prediction | |
| --- | --- | --- | --- | --- | --- |
|  |  | Prediction | Score | Prediction | Score |
| rs5985 | Nonsynonymous | Tolerated | 1 | Benign | 0 |
| rs5982 | Nonsynonymous | Tolerated | 0.14 | Benign | 0.003 |

**Table S2.**Prediction of functional effect of studied SNPs
